# Advancing breast cancer rehabilitation: a novel tool for assessing physical morbidity risk

**DOI:** 10.1093/oncolo/oyaf060

**Published:** 2025-05-14

**Authors:** Ifat Klein, Merav A Ben David, Danit R Shahar, Irena Rosenberg, Sergio Susmallian, Daphna Barsuk, Michael Friger

**Affiliations:** Assuta Medical Center, Ramat Hahayal, Tel Aviv 6971028, Israel; Department of Epidemiology, Biostatistics and Community Health, School of Public Health, Faculty of Health Sciences, Ben-Gurion University of the Negev, Beer Sheva 8410501, Israel; Assuta Medical Center, Ramat Hahayal, Tel Aviv 6971028, Israel; Faculty of Health Sciences, Ben-Gurion University of the Negev, Beer Sheva 8410501, Israel; Department of Epidemiology, Biostatistics and Community Health, School of Public Health, Faculty of Health Sciences, Ben-Gurion University of the Negev, Beer Sheva 8410501, Israel; Assuta Medical Center, Ramat Hahayal, Tel Aviv 6971028, Israel; Assuta Medical Center, Ramat Hahayal, Tel Aviv 6971028, Israel; Assuta Medical Center, Ramat Hahayal, Tel Aviv 6971028, Israel; Department of Epidemiology, Biostatistics and Community Health, School of Public Health, Faculty of Health Sciences, Ben-Gurion University of the Negev, Beer Sheva 8410501, Israel

**Keywords:** breast cancer, risk factors, morbidity, physical impairments, risk assessment, tool development, rehabilitation, physical therapy

## Abstract

**Purpose:**

This study presents the development process of the Arm Morbidity following Breast Cancer Treatments (ARM-BCT) tool.

**Materials and methods:**

A historical prospective study was conducted across five medical centers from 2020 to 2023. Medical information and a questionnaire covering morbidities, lifestyle, emotional state, and functioning were collected. Regression models analyzed 22 risk factors for chronic pain, lymphedema, functional limitations, and decreased range of motion. Significant factors were included in the ARM-BCT tool.

**Results:**

Seventeen significant risk factors were identified, including mastectomy (*B* = 2.073, CI, 7.403–5.366), axillary lymph node dissection (*B* = 0.194, CI, 0.988–1.036), breast reconstruction (*B* = 17.300, CI, 7.105–27.495), advanced stage (*B* = 0.498, CI, 1.044–2.594), chemotherapy (*B* = 1.326, CI, 0.870–3.673), BMI (*B* = 0.092, CI, 1.033–1.163), anxiety (*B* = 0.177, CI, 1.859–3.079), low physical activity levels (*B* = -0.059, CI, 0.190–0.001), specific comorbidities (*B* = -1.491, CI, 2.706–0.277), age (*B* = 0.035, OR = 1.036, CI, 1.002–1.071), and radiation therapy (*B* = 0.385, CI, 0.380–2.056), etc. The tool’s development involved robust statistical modeling to determine the weight of each factor, evaluate model quality, and establish a clinically relevant cutoff point.

**Conclusion:**

This article describes the development process of the ARM-BCT tool, designed to assess the risk of physical morbidity following breast cancer treatment. The tool incorporates 17 statistically significant risk and protective factors into a scoring scale ranging from 1 to 20. Risk is categorized as low (< 6) or high (> 7), enabling targeted recommendations for rehabilitation timing and necessity. While validation studies evaluating its clinical effectiveness are underway and will be presented in future publications, the ARM-BCT tool shows promise in enhancing recovery outcomes through early intervention.

**Implications for practice**

This study reviewed both risk and protective factors that influence recovery following breast cancer surgery and treatments. The significant factors identified were incorporated into the newly developed ARM-BCT tool, designed to assess the risk of upper limb morbidity during cancer treatment.

The ARM-BCT tool can be utilized by a range of healthcare professionals, including primary care physicians, oncologists in oncology clinics, discharge nurses following surgery, and physical or occupational therapists. Importantly, the tool is also accessible and user-friendly for patients themselves.

The tool comprises 17 contributing factors, of which 5 factors—emotional state, family support, sleep disturbances, and physical activity levels—are self-reported by the patient. The remaining 12 factors can be obtained from the patient’s medical records.

The tool employs a cut-off score of 7, classifying patients into high-risk and low-risk groups. This stratification allows for tailored recommendations and targeted referrals to rehabilitation services. Early, preventive, or proactive interventions aligned with the patient’s specific risk profile have the potential to improve physical recovery outcomes.

The ARM-BCT tool has undergone additional validation to assess its predictive accuracy. While the results have not yet been published, the analyses suggest its potential in identifying individuals at higher risk of arm morbidity following breast cancer surgery.

## Introduction

Breast cancer (BC) is the most prevalent cancer among women worldwide, accounting for 25.8% of the total new cases diagnosed in 2020.^1^ Ranking first in newly diagnosed cases among women in the United States,^2^ with over 3.5 million women having experienced invasive BC.^3^ Understanding and addressing the diverse treatments for this disease is of paramount importance.

An array of BC treatments, ranging from surgery to various therapeutic modalities, such as radiation, chemotherapy, biological, hormonal, and immunotherapy,^4^ often include a wide spectrum of side effects. These side effects, both short- and long-term, can significantly impact the mental–emotional, cognitive, functional, and physical well-being of patients.^5^

The Arm Morbidity following BC Treatments (ARM-BCT) tool was designed to specifically address the physical-functional dimension of these patients with the aim of encompassing the full spectrum of morbidities that women may encounter, including persistent pain, lymphedema, diminished range of motion (ROM) and reduced functionality.

Lymphedema, a challenging and often debilitating condition, typically emerges within months to years following surgery and afflicts around 15%-25% of women who underwent axillar lymph node dissection (ALND) and approximately 6% of those undergoing sentinel lymph node biopsy (SLNB).^6^ Lymphedema results in physical impairments, including compromised functionality, reduced strength, and discomfort of the affected arm.^7,8^ Notably, persistent pain affects 30%–50% of women, while limitations in arm and shoulder mobility have been self-reported by approximately 35% of patients, often persisting for years after the surgical intervention.^9,10^

Prolonged arm morbidity following breast and ALND surgery exerts a profound impact on patients’ activities of daily living (ADL), physical activity (PA) engagement,^11^ quality of life (QoL),^12^ and overall health.^13^ Moreover, individuals grappling with arm morbidity often encounter difficulties in returning to work and experience a decline in their functional abilities.^14^ These challenges can delay BC survivors from regaining the lives and routines in which they participated prior to their cancer diagnosis.^15^

The identification of patients at a higher risk of future arm morbidity and the provision of personalized recommendations or early-stage interventions shows the potential to significantly alleviate long-term morbidity.^16,17^ Intriguingly, such a tool has not yet been developed, and this article presents a comprehensive account of the research design aimed at its creation.

### Material and methods

A convenience sample was used in this retrospective-prospective study conducted across five medical centers between October 2022 and March 2023. Women aged 18-75 years, functionally independent, and who underwent various types of oncological breast surgeries were included. Participants were recruited between 6–36 months post-surgery to evaluate long-term morbidity.

Exclusion criteria comprised patients with advanced metastatic disease, women who underwent non-oncological surgeries (eg, fibroadenoma excision or unrelated plastic surgeries), small procedures such as breast biopsy, and individuals reporting upper limb lymphedema predating surgery.

The study received Institutional Review Board approval under protocol number ASMC-0019-22. Digital informed consent was obtained from all participants prior to completing the study questionnaire.

The data collection process involved the acquisition of information from hospital computer systems, which included details about the type of surgery, socio-demographic characteristics, and medical variables, as well as surgical and pathology reports. Additionally, data was collected through a digital questionnaire sent to patients via a link to their mobile phones.

Outcome measures included quick disabilities of the arm, shoulder, and hand (Quick DASH) questionnaire,^18^ Numeric pain rating scale (NPRS),^19^ self-reporting limitation abduction ROM, self-reported diagnosis of lymphedema made by a physician or physical therapist, emotional well-being using SF 36^20^ and PA level using Godin–Shephard Leisure-Time.^21^

### Statistical analysis

The sample size was determined by assessing the incidence of morbidity and functional decline associated with SLNB and ALND procedures.^22^ Functional decline was evaluated using Quick DASH in SLNB patients, with a mean of 14.0 ± 16.2 (*n* = 506), and ALND patients, mean score 18.1 ± 15.9 (*n* = 255),^23^ aiming for a type I error probability of 0.05 and a type II error probability of 0.2, a sample size of 378 patients per group was calculated. Given that both morbidity-present and morbidity-absent groups were assessed, the study required a total sample of 756 participants.

Statistical analyses were carried out using the SPSS statistical package, Version 21 (SPSS Inc.). Categorical variables were summarized in terms of frequencies and percentages, while continuous data were presented as means along with their respective standard deviations (SD). For each type of morbidity (chronic pain, lymphedema, limitation of ROM, and decrease in function), a separate statistical model was established. Initially, potential univariate relationships with a variety of different risk factors were examined. After examining collinearity, confounding variables, and interactions, multivariate models using various regression techniques were created.

Significant risk factors, were identified in each model and then incorporated into the newly developed tool, and their significance (*P*) in addition to beta 95% associated odds ratio (OR) values and confidence interval (CI) were comprehensively reported. This approach ensured that the selected risk factors were incorporated into the tool with appropriate consideration of their individual contribution to morbidity risk assessment. Additional analyses were conducted to determine the weights of the risk factors included in the tool and establish the cut-off points for categorizing low and high risk of morbidity.

## Results

### Study participant recruitment and inclusion

**Figure 1.**
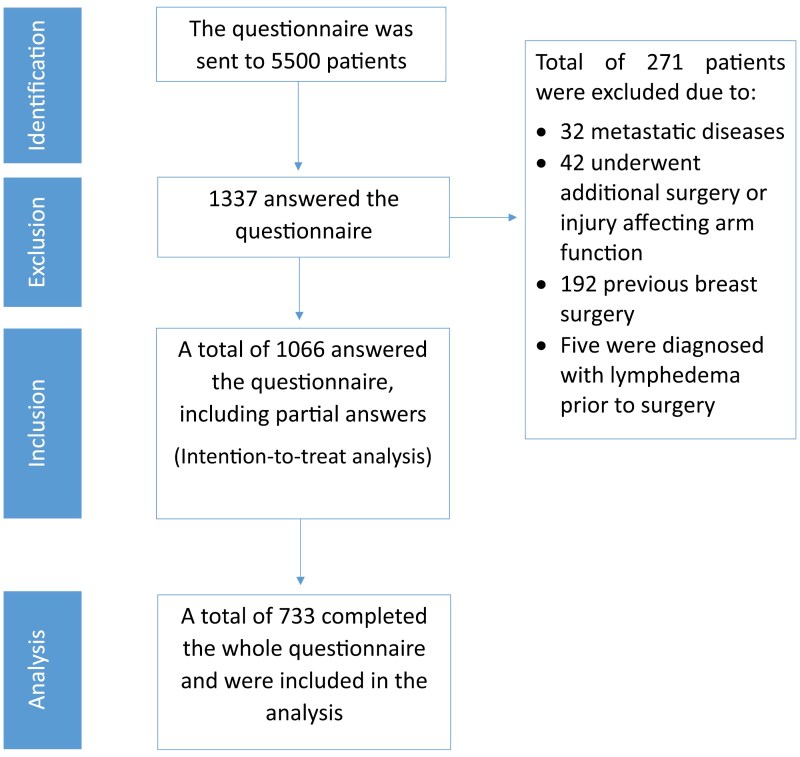
Study flow chart consolidated standards of reporting trials (CONSORT) diagram.

A digital questionnaire was distributed to 5500 patients, out of the recipients, 1337 (24.3%) filled out and returned the questionnaire. To minimize bias associated with the convenience sampling method, the personal and sociodemographic characteristics of women who completed the questionnaire were compared with those who did not. The only observed difference was that among the women who agreed to participate, were younger (56.0 ± 13.8) compared to the 3663 women who did not participate in the study (54.16 ± 14.4), with a p-value of 0.001. Of the remaining patients 271 (20.2%) patients were excluded based on predetermined exclusion criteria, leaving 1066 patients eligible for inclusion in the subsequent statistical analyses. The CONSORT diagram illustrating the inclusion process is shown in Figure 1.

Given the sensitive nature of the questionnaire for patients treated for or recovering from BC, some questions were optional, leading to varying response rates across different sections. To ensure comprehensive analysis, an intention-to-treat approach was adopted, including partially completed questionnaires. The response rates for different questions are reported for each model, with 733 patients (68.9%) completing the entire questionnaire.

### Demographic and arm morbidity characteristics

The study sample included patients with a mean age of 56.9 years (SD ± 11.6) and a mean body mass index (BMI) of 26.5 kg/m² (SD ± 12.8). The mean time elapsed since surgery was 24.0 months (SD ± 11.6). The characteristics of the patients, type of therapy, and lifestyle of patients with and without morbidity are detailed in Table 1.

**Table 1.** Patients, type of therapy, and lifestyle characteristics.

	Total	No morbidity		With morbidity		*P*
Continues factors	Mean ± SD	*N*	Mea*n* ± SD	*N*	Mean ± SD	
Categorical factors		*N* (%)		*N* (%)		
***Personal factors***						
BMI	26.5 ± 12.8	155	26.4 ± 5.4	733	26.2 ± 5.0	0.875
Age	56.9 ± 11.6	156	58.4 ± 12.8	742	56.9 ± 12.1	0.074
Family status	Single	15 (10.6%)		76 (10.8%)		0.712
	Married	101(71.6%)		516 (73.1%)		
	Single parent	1 (0.7%)		1 (0.1%)		
	Divorcee	18 (12.8%)		89 (12.6%)		
	Widow	6 (4.3%)		23 (3.3%)		
	Separated	0		1 (0.1%)		
Stage	Pre-cancerous	85 (57.0%)		193 (26.4%)		**<0.001**
	Local	56 (37.65%)		474 (64.8%)		
	Advance	8 (5.4%)		65 (8.9%)		
Economic status	Below average	6 (4.4%)		18 (2.5%)		0.339
	Average	54 (39.7%)		253 (34.6%)		
	Good	51 (37.5%)		313 (42.8%)		
	Very good	25 (18.4%)		147 (20.1%)		
Educational Level	High school	51 (36.7%)		203 (27.4%)		**0.010**
	BA	56 (40.3%)		254 (34.7%)		
	Master	20 (14.4%)		160 (21.9%)		
	PHD≤	12 (8.6%)		114 (15.6%)		
BRCA carrier	26 (16.7%)	128 (17.3%)		0.844		
Comorbidity^*^		408 (34.0%)		53 (20.2%)		**<0.001**
Smoking		18 (13.2%)		86 (11.8%)		0.628
***Surgery related factors***						
Type of surgery	Lumpectomy Mastectomy	126 (80.8%) 17 (11.1%)		599 (81.6%) 123 (16.8%)		**0.001**
	Prophylactic	10 (6.5%)		12 (1.6%)		
Extreme pain during hospitalization (yes)		18 (1.5%)		139 (18.7%)		**0.032**
Breast reconstruction	No reconstruction	137 (87.8%)		630 (84.9%)		0.530
	Autologous	6 (3.9%)		37 (4.9%)		
	Implants	13 (8.3%)		75 (10.1%)		
ALND	2.5 ± 3.3	156	1.8 ± 2.0	742	2.8 ± 3.7	**0.001**
Post-operative complications	No	99 (86.1%)		614 (84.9%)		0.763
	Bleeding	2 (1.7%)		27 (3.7%)		
	Revision surgery	5 (4.3%)		22 (3.0%)		
	Seroma	9 (7.8%)		1 (0.1%)		
Axillar web syndrome		4 (2.6%)		58 (7.8%)		**0.019**
***Treatment-related factors***						
Neoadjuvant chemotherapy		12 (7.7%)		110 (14.8%)		**0.018**
Adjuvant chemotherapy		13 (8.3%)		115 (15.5%)		**0.020**
Radiation to the breast		71 (45.5%)		487 (65.6%)		**<0.001**
Radiation to the axilla		16 (10.3%)		173 (23.3%)		**<0.001**
Hormonal treatment		34 (21.8%)		238 (32.1%)		**0.011**
***Physical therapy***						
Received PT during hospitalization		23 (32.9%)		306 (43.1%)		0.098
Not received physical therapy after surgery		116 (78.4%)		306 (41.2%)		**<0.001**
***Physical activity***						
Physical activity level	10.1 ± 13.8	156	8.6 ± 10.7	742	12.5 ± 14.9	**0.001**
***Emotional state***						
Emotional well being	24.3 ± 5.1	64	25.8 ± 3.6	712	24.5 ± 5.2	0.056
Anxiety level	2.0 ± 3.0	115	1.3 ± 1.4	720	2.1 ± 3.0	0.078
No family support		7 (6.3%)		57 (7.9%)		0.536
Insomnia		20 (17.5%)		390 (54.0%)		**<0.001**

Abbreviations: BMI, body mass index; BRCA, breast cancer susceptibility gene, PT, physical therapy, SD, standard deviation , ALND, axillar lymph node dissection. Bold *P* values indicate statistical significance (*P* < 0.05).

^*^Comorbidities included orthopedic and neurological issues, diabetes, hypertension, respiratory, cardiac, and renal failure.

The average Quick DASH score was 5.5 ± 22.1, the NPRS score was 2.6 ± 2.8, depression score (0-10) was 3.5 ± 2.3, and anxiety score (0-10) was 2.1 ± 3.0. Additionally, 299 participants (37.5%) self-reported limitations in range of motion (ROM), and lymphedema self-reported by 60 participants (7.5%).

The development of the Arm Morbidity following Breast Cancer Treatments tool

The tool was developed through the following steps:

#### 1. Identifying the risk and beneficial factors:

By finding statistical relationships between the 22 different risk factors was tested in four different models, one for each common morbidity of the arm.

### Model 1—Risk factor analysis for function disability (Quick DASH)

In the quantile regression, that included 971 patients (91.0%) where various risk factors were identified, including age over 58 (95% CI, 0.014; 0.079), advanced disease stage (95% CI, 3.292; 0.855), poor emotional state (95% CI, 1.369; 0.598), anxiety (95% CI, 1.859; 3.079), pain over 6 during hospitalization (95% CI, 10.777; 19.733), ALND (95% CI, 0.988; 1.036), and mastectomy (95% CI, 7.403; 5.366). The full CIs of each significant risk factor are presented in Table 2.

**Table 2.** Confidence interval (95%) of significant risk factors for functional disability by Quick DASH divided into quantiles.

Quantiles / risk factors	*0.10*	*0.25*	*0.5*	*0.75*	*0.9*
Age > 58	0.014; 0.079				
Advanced stage	3.292; 0.855				
Low emotional well-being	0.330; 0.074	0.330; 0.074	0.736; 0.310	1.293; 0.687	1.369; 0.598
Low physical activity level	0.099; 0.019	0.099; 0.019		0.190; 0.001	
High anxiety level	0.060; 0.293	0.292; 0.698	0.542; 1.218	1.197; 2.157	1.859; 3.079
Comorbidity^*^		2.706; 0.277			
Extreme pain during hospitalization	0.460; 2.634	1.162; 4.946	7.229; 13.536	10.777; 19.733	
No PT after surgery	2.766; 1.233	4.259; 1.591	6.464; 2.017	7.225; 0.911	11.941; 3.910
No Family support	2.532; 0.129	4.593; 0.410			
Insomnia			1.238; 6.214	1.548; 8.614	6.986; 15.973
PO complications^**^				0.074; 2.434	
ALND	0.103; 0.285				0.988; 1.036
Mastectomy surgery	3.292; 0.855				7.403; 5.366
Reconstruction surgery					7.105; 27.495

Abbreviations: ALND, axillar lymph node dissection, PT, physical therapy; PO, post-operative).

^*^Comorbidities included neurological problems, orthopedic issues, and fibromyalgia and/or chronic pain.

^**^Post-operative complications included bleeding, infection, revision surgery, and/or seroma.

### Model 2—Risk factor analysis for prolonged pain (NPRS)

The regression included 709 patients (66.5%) in which various risk factors were identified, including chemotherapy (95% CI, 0.870; 3.673), radiation (95% CI, 0.380; 2.056), anxiety (95% CI, 0.202; 0.504), pain score higher than 6 on NPRS during hospitalization (95% CI, 3.184; 6.428), and women who did not receive physical therapy during hospitalization (95% CI, 2.228; 1.260). The full CI results are shown in Table 3.

**Table 3.** Confidence interval (95%) of significant risk factors for prolonged pain by Numeric Pain Rating Scale divided into quantiles.

Quantiles / risk factors	*0.10*	*0.25*	*0.5*	*0.75*	*0.9*
Advanced stage	0.092; 0.432				
Low emotional well-being			0.092; 0.041	0.218; 0.110	0.244; 0.083
High anxiety level	0.019; 0.067	0.087; 0.009	0.102; 0.199	0.248; 0.450	0.202; 0.504
Comorbidity^*^					2.80; 0.554
Extreme pain in hospitalization			3.184; 6.428		
No PT after surgery	0.069; 0.482		0.708; 0.243	2.228; 1.260	2.164; 0.721
ALND	0.057; 0.009	0.047; 0.019	060; 0.014		
Reconstruction surgery	0.026; 0.417				0.103; 1.467
Adjuvant chemotherapy		0.067; 2.585	0.870; 3.673		
Radiation to axilla			0.115; 0.655	0.037; 1.161	0.380; 2.056

Abbreviations: ALND, axillar lymph node dissection; PT, physical therapy.

^*^Comorbidities included neurological problems, orthopedic issues, and fibromyalgia and/or chronic pain.

### Model 3—Risk factor analysis for self-reported range of motion limitations

The risk factors were examined using logistic regression. The model included 734 female participants (68.9%). It was found that metastatic disease (OR 1.654), extreme pain during hospitalization (OR 3.541), poor emotional well-being (OR 0.850), and absence of PT during hospitalization (OR 1.576) were significant risk factors, as shown in Table 4.

**Table 4.** Significant risk factors associated with shoulder range of motion limitations and with lymphedema development.

Risk factors	*P*	*B*	OR	95% CI
Factors associated with shoulder range of motion limitations				
Advanced stage	0.032	0.498	1.645	1.044; 2.594
Extreme pain during hospitalization	< 0.001	1.264	3.541	1.792; 6.995
Low emotional well-being	< 0.001	0.162	0.850	0.794; 0.910
No PT after surgery	< 0.001	0.979	1.576	0.252; 0.561
Factors associated with lymphedema development				
Age > 58 years	0.039	0.035	1.036	1.002; 1.071
BMI > 27	0.003	0.092	1.096	1.033; 1.163
ALND	<0.001	0.110	1.116	1.055; 1.180
High anxiety level	<0.001	0.220	1.246	1.115; 1.392
No PT after surgery	<0.001	2.481	0.084	0.030;0.230

Abbreviations: ALND, axillar lymph node dissection; *B*, beta; BMI, Body mass index; CI, 95% confidence interval; OR, odds ratio; *P*, sig; PT, physical therapy; QOL, quality of life..

### Model 4—Risk factor analysis for self-reported lymphedema development

The risk factors were examined using logistic regression, with 759 participants (71.2%) included in the model. It was found that age over 58 (OR 1.036), BMI over 27 (OR 1.096), and ALND (OR 1.116) were significant risk factors, as shown in Table 4.

### The impact of comorbidities: identifying influential medical conditions

In the questionnaire sent to participants, comorbidities such as heart failure, respiratory problems, kidney problems, and hypertension were examined. Additional analysis revealed that neurological problems, orthopedic issues, and fibromyalgia were significantly associated with functional limitations (*P* = 0.013, *P* = 0.001, and *P* = 0.013, respectively) and ROM limitations (*P* = 0.001 each). Diabetes and neurological problems were linked to lymphedema (*P* = 0.010 and *P* = 0.017, respectively). Low hemoglobin and high blood pressure were related to prolonged pain (*P* = 0.040 and *P* = 0.004, respectively). Due to confounding factors, diabetes, low hemoglobin, and high blood pressure were excluded from the new tool. Neurological problems, orthopedic issues, and fibromyalgia were included in the comorbidity section of the ARM-BCT tool.

#### Estimating the weight of each risk factor

A dichotomous morbidity variable was used (scoring 0 for no morbidity and 1 for the presence of one or more of the four diseases being tested). In the model, all 17 variables included in the tool were tested. The beta coefficients of each variable were calculated to estimate the weights of the significant variables. The factors found to be significant were ALND (*B* = 0.944, *P* = 0.020, OR = 0.058) and postoperative complication (*B* = 0.160, *P* = 0.045, OR = 0.852). The model had an ROC curve area under the curve of 0.840, *P* < 0.001, 95% CI, 0.773;0.908.

#### Adjusting the various risk factors to design them into a questionnaire format

This involved refining measurement methods and scoring criteria across different sections.

The level of PA categorization distinguishes between high intensity (−1), light activity (0), and no PA (1). The emotional well-being section, originally evaluated by the SF-36 questionnaire, was streamlined to a single question on depression, rated on a scale of 0-10, and the cutoff score was identified.

#### Developing the algorithm for calculating overall risk

All 17 variables were integrated into the questionnaire. The algorithm for calculating the risk was based on the weight and structure of the questions. The total risk score was then calculated by summing these weighted scores, providing a comprehensive risk assessment for arm morbidity following BC treatments. The developed questionnaire had a value range of 1-20.

#### Determining the risk for arm morbidity cutoff for the new tool

The determination of the morbidity risk threshold was performed for the RAM-BCT tool in relation to a dichotomous morbidity variable. The new tool assigns a score ranging from 1-20 for the risk of developing long-term arm morbidity. To establish the cutoff score for high risk, a Youden’s statistic test was performed, resulting in an identified cutoff of 6.5.^24^ The area under the receiver operating characteristic (ROC) curve (AUC) of 0.841 (95% CI, 0.815–0.866) demonstrates the model’s good performance in distinguishing between high- and low-risk individuals.

The summary of risk factors by morbidity is presented in Table 5 and Supplementary Figure S1.

**Table 5.** Summary of the factors found to be significant in the four physical ailments examined.

Morbidity	Decreased Function	Prolonged pain	Decreased ROM	Lymphedema
Risk factors	**Age** (*P* = 0.005, *B* 0.046, CI 0.014; 0.079) **Mastectomy type of surgery** (*P* < 0.001, *B* 2.073, CI 7.403; 5.366) **Stage** (*P* = 0.029, *B* -0.675, CI, 3.292; 0.855) **ALND** (*P* < 0.001, *B* 0.194, CI, (0.988; 1.036) **Anxiety **(*P* = 0.003, *B* 0.177, CI, 1.859; 3.079) **Extreme pain during hospitalization** (*P* = 0.005, *B* 1.547, CI, 10.777; 19.733) **No PT after surgery** (*P* < 0.001, -*B* 1.999, CI, 4.593; 0.410) **No Family support** (*P* = 0.030, -*B* 1.330, CI, 4.593; 0.410) **Low emotional well-being** (*P* = 0.002, *B* 0.202 CI, 369; 0.598) **PA level** (*P* = 0.004, *B* -0.059, CI, 0.190; 0.001) Comorbidity^*^ (P = 0.016, B -1.491, CI 2.706; 0.277) **Insomnia** (*P* = 0.003, *B* 3.726, CI, 6.986; 15.973) Postoperative complications^**^ (*P* = 0.037, *B* 1.254, CI, 0.074; 2.434) **Breast reconstruction** (*P* < 0.001, *B* 17.300, CI, 7.105; 27.495)	**Breast reconstruction** (*P* = 0.026, *B* 0.221, CI, 0.103; 1.467) **Stage** (*P* = 0.003, *B* 0.262, CI, 0.092; 0.432) **No PT after surgery** (*P* = 0.009, *B* 0.275, CI, 2.228; 1.260) **Adjuvant chemotherapy** (*P* = 0.039, *B* 1.326, CI, 0.870; 3.673) **Low emotional well-being** (*P* = < 0.001, *B* 0.067, CI, 0.244; 0.083) **Anxiety** (*P* < 0.001, *B* 0.151, CI, 0.248; 0.450) **Extreme pain during hospitalization** (*P* < 0.001, *B* 4.788, CI, 3.184; 6.428) **Radiation to axilla** (*P* = 0.005, *B* 0.385, CI, 0.380; 2.056) Comorbidity* (*P* = 0.003, *B* -1.678, CI, 2.803; 0.554) **ALND** (*P* < 0.001, -*B* 0.024, CI, 060; 0.014)	**Extreme pain during hospitalization** (*P* = < 0.001, *B* 1.264, OR 3.541, CI, 1.792; 6.995) **Low emotional well-being** (*P* = < 0.001, *B* 0.162, OR 0.850, CI, 0.794; 0.910) **No PT after surgery** (*P* = < 0.001, *B* 0.979, OR 1.576, CI, 0.252; 0.561). **Stage **(*P* = 0.032, *B* 0.498, OR 1.645, CI, 1.044; 2.594)	**Age** (*P* = 0.039, *B* 0.035, OR 1.036, CI, 1.002; 1.071) **BMI** (*P* = 0.003, OR 1.096, *B* 0.092, CI, 1.033; 1.163) **ALND** (*P* = < 0.001, OR 1.116, *B* 0.110, CI, 1.055; 1.180) **Anxiety **(*P* = < 0.001, OR 1.246, *B* 0.220, CI, 1.115; 1.392) No PT after surgery^*^ (P = <0.001, B -2,481, OR 0.084, CI, 0.030; 0.230)

Abbreviations: ALND, axillar lymph node dissection; *B*, beta; CI, 95% confidence interval; OR, odds ratio; *P*, sig; PT, physical therapy; ROM, range of motion. Confidence interval of the highest of all quantiles in each category is reported.

^*^Comorbidities included neurological problems, orthopedic issues, and fibromyalgia and/or chronic pain.

^**^Post-operative complications included bleeding, infection, revision surgery, and/or seroma.

#### Validation and effectiveness evaluations

Validation, refinement, and final verification of the tool in the second study (ASMC 0018-23), part of a series of studies for its development, with the third study focusing on implementation and effectiveness testing, both currently in their final stages.

## Discussion

BC patients may experience a range of physical ailments, including pain,^25^ ROM limitations,^23^ decreased function,^9,26^ and lymphedema,^27^ which can develop weeks to months after surgery and oncological treatments and persist sometimes for months to years.^10^

Rehabilitation strategies for BC patients play a pivotal role in enhancing their QoL.^28,29^ They encompass efforts to improve a patient’s ROM,^30^ enhance strength, and reduce pain.^31^ Additionally, complete decongestive therapy serves as a conservative treatment for lymphedema.^32^ Following breast surgery, rehabilitation serves to facilitate functional recovery,^33^ and encourage patients to resume PA and work.^34^ During oncological treatment, physical rehabilitation and exercise were found to mitigate side effects and potentially enhance survival rates while reducing the risk of disease recurrence.^35,36^ Given these considerations, models like the prospective follow-up model have been developed to actively monitor, identify, and address physical impairments and functional limitations.^33,37^ These approaches are grounded in extensive knowledge about the effectiveness of physical therapy, occupational therapy, and various types of PA in treating physical morbidity.^38,39^

To enhance the identification of patients at risk of morbidity and promote women’s health by referring patients to various rehabilitation programs, before the development of symptoms this tool was developed.^40,41^ The construction process of the ARM-BCT tool was systematically conducted to identify all the risk factors that could cause common physical ailments. For each condition, different statistical models were tested, and out of the 22 risk factors found in the literature,^42^ 17 significantly influencing factors were identified. These risk factors were divided into six categories: personal risk factors (eg, age > 57, BMI > 27, comorbidities including orthopedic, neurological, or chronic pain conditions); factors related to surgery (eg, type of surgery, ALND, high pain level during hospitalization, and postoperative complications); factors related to oncology treatment (eg, disease stage, chemotherapy, and radiation therapy); and emotional factors (eg, sleep difficulties, anxiety, depression and lack of family support). Additionally, two beneficial factors were identified: a high level of PA before surgery and receiving training for exercises from a physical therapist during hospitalization.

In further depth, the various comorbidities were examined to understand more precisely which factors adversely affect physical recovery. In this study, neurological conditions, orthopedic injuries, and chronic pain, including fibromyalgia, were identified as factors that adversely impact lymphedema, functional decline, and reduced ROM. No similar relationship was found with chronic pain, as noted by Doan et al., who reported a relationship between old age and comorbidities, including back pain, arthritis or arthrosis, fibromyalgia, and neck pain, in addition to frozen shoulder, sciatica, migraine, and systemic lupus.^43^

The construction of the tool relied on various studies for the development of questionnaires and risk assessment tools,^44-47^ and followed a systematic approach: (1) identification of all risk factors, (2) developing a suitable question for each risk factor and beneficial factor, (3) calculating the weights of the various factors, (4) distributing the score to each factor based on its weight, and (45) building the formula for calculating the risk.

The factors were summarized to create a short and easy-to-use questionnaire. Therefore, the hope is that the application of the tool will be easy and convenient, leading to frequent use in breast units, oncology departments, including breast and radiation units in hospitals, and by treating doctors. Moreover, patients themselves can employ the tool for risk assessment, fostering a patient-centred approach through the use of patient-reported outcome measures (PROMs).^48^ The effectiveness of the tool and the provision of proactive treatment immediately after surgery are being evaluated in additional studies.

Several different tools have been developed for the purposes of identifying lymphedema, decreased QoL, and chronic pain. The Morbidity Screening Tool focuses on evaluating fatigue, pain, swelling (lymphedema), and arm function in the chemotherapy phase.^49^ Other tools concentrate on assessing the risk of lymphedema development and include a risk factor-based scoring system^50^ These include the Lymphedema and BC questionnaire,^51^ the Upper Limb Lymphedema questionnaire,^52^ lymphedema Life Impact Scale, the Upper Limb Lymphedema 27 scale^45^ and the Norman Lymphedema Survey.^53^ The Functional Assessment of Cancer Therapy plus 4 has been developed to assess the QoL of BC patients.^54^ Other tools are used to measure chronic pain.^55^ The development of the new tool was motivated by the goal of identifying the combination of common physical ailments that women suffer from after oncological surgeries and treatments, including prolonged pain, lymphedema, decreased ROM, and decreased function. These conditions usually affect patients’ QoL, and paramedical professionals have tools to help treat and alleviate.

The study has several limitations related to data collection: (1) it relied solely on a questionnaire rather than objective evaluations, such as measuring ROM and arm circumferences, (2) the questionnaire’s design allowed for non-obligatory responses, which led to varying response rates across different questions and potentially affected the strength of the statistical analysis.

However, the study’s strengths lie in its extensive inclusion of female patients from five hospitals across diverse geographical areas who were treated by a variety of surgeons using contemporary techniques and up-to-date treatments. Furthermore, the study’s foundation which was based on a preliminary literature review facilitated a systematic examination of various risk factors from different domains, including surgical procedures, treatments, PA levels, specific comorbidities, and emotional support in conjunction with emotional well-being.

## Conclusion

The ARM-BCT tool emerged from a comprehensive study examining the influence of diverse risk factors across various domains on the occurrence of physical arm morbidity and included issues, such as prolonged pain, restricted ROM, diminished function, and lymphedema. Utilizing a 17-item assessment with a score ranging from 1 to 20, the ARM-BCT is designed to assist in identifying personalized risk of morbidity. Identifying women at risk and offering adapted rehabilitation interventions or follow-up treatment may contribute to reducing morbidity rates and improving recovery following surgery and oncological treatments.

## Supplementary Material

oyaf060_suppl_Supplementary_Figures_S1

## Data Availability

The data collected during this study will be available upon request in a coded format to ensure participant confidentiality. Requests for data can be made at any stage of the research process.
