# Supplementary material for: Advancing breast cancer rehabilitation: a novel tool for assessing physical morbidity risk
Source: Oncologist. 2025 May 14;30(5):oyaf060. doi: 10.1093/oncolo/oyaf060 (PMC12159735; doi:10.1093/oncolo/oyaf060)
Supplement: oyaf060_suppl_Supplementary_Figures_S1 [file oyaf060_suppl_supplementary_figures_s1.zip › oyaf060_suppl_Supplementary_Figures_S1.pdf]

| Category | Factor | Values |
| --- | --- | --- |
| **Personal factors** | **Age** | < 57 > 58 |
| | **BMI** | < 26 > 27 |
| | **Comorbidity** Orthopedic/ neurological problems/ fibromyalgia | No, Yes |
| **Physical activity** | **Physical activity** | Sedentary, moderately active and Active |
| **Surgery related factors** | Surgery | Lumpectomy, Mastectomy |
| | **Breast reconstruction** | No, Yes |
| | **Number of dissected lymph nodes** | Non, 1-4 nodes, more than 5 |
| | **Extreme Pain during hospitalization** | No, Yes |
| | **Stage** | Precancerous or localized (0 Ⅰ Ⅱ) Advanced or metastatic (Ⅲ) |
| | **Post operative complications** | No, Yes |
| **Treatment related factors** | **Chemotherapy** | No, Yes |
| | **Radiation treatment** | No, Yes |
| **Physical therapy** | **Physical therapy during hospitalization** | No, Yes |
| **Emotional state** | **Anxiety 0-10** | ≤3 4≤ |
| | **Insomnia or difficult sleeping** | No, Yes |
| | **Depression 0-10** | ≤3 4≤ |
| | **Family support** | No, Yes |
