# Supplementary material for: Advancing breast cancer rehabilitation: a novel tool for assessing physical morbidity risk
Source: Oncologist. 2025 May 14;30(5):oyaf060. doi: 10.1093/oncolo/oyaf060 (PMC12159735; doi:10.1093/oncolo/oyaf060)
Supplement: oyaf060_suppl_Supplementary_Figures_S1 [file oyaf060_suppl_supplementary_figures_s1.zip › oyaf060_suppl_Supplementary_Figure_S1.docx]

## Supplementary Data

**Supplementary Figure S1.** The 17 factors incorporated in the ARM-BCT.


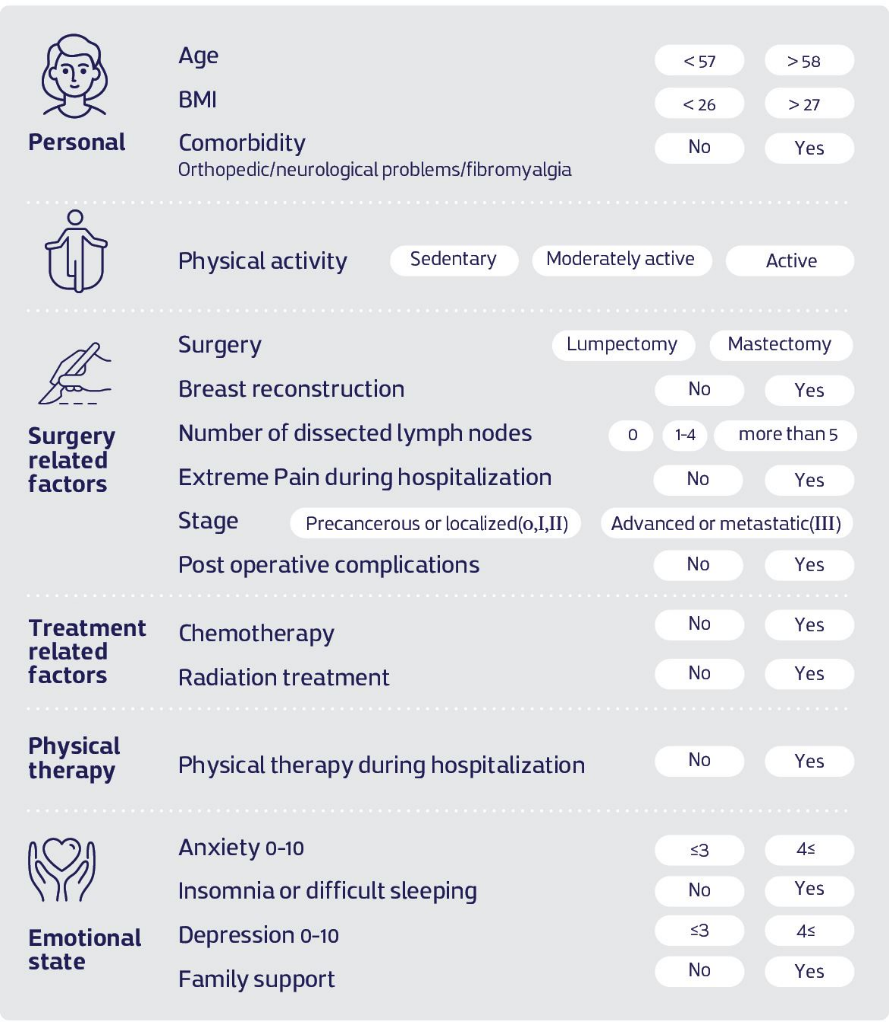


This figure presents the 17 factors incorporated into the ARM-BCT, which is designed to estimate the risk for impaired physical recovery of the arm following breast cancer treatment. The factors are organized into four main domains: personal risk factors, surgical factors, oncology treatment factors, and emotional/psychosocial factors.
Additionally, the figure highlights two protective factors associated with improved recovery: engaging in high levels of physical activity prior to surgery and receiving exercise guidance from a physical therapist during hospitalization.
